# ER-Stress and Senescence Coordinately Promote Endothelial Barrier Dysfunction in Diabetes-Induced Atherosclerosis

**DOI:** 10.3390/nu14142786

**Published:** 2022-07-06

**Authors:** Sameen Fatima, Saira Ambreen, Akash Mathew, Ahmed Elwakiel, Anubhuti Gupta, Kunal Singh, Shruthi Krishnan, Rajiv Rana, Hamzah Khawaja, Dheerendra Gupta, Jayakumar Manoharan, Christian Besler, Ulrich Laufs, Shrey Kohli, Berend Isermann, Khurrum Shahzad

**Affiliations:** 1 Institute of Laboratory Medicine, Clinical Chemistry and Molecular Diagnostic, University Hospital, 04103 Leipzig, Germany; sameen.fatima@medizin.uni-leipzig.de (S.F.); saira.ambreen@medizin.uni-leipzig.de (S.A.); akash.mathew@medizin.uni-leipzig.de (A.M.); ahmed.elwakiel@medizin.uni-leipzig.de (A.E.); anubhuti.gupta@medizin.uni-leipzig.de (A.G.); kunal.singh@medizin.uni-leipzig.de (K.S.); shruthi.krishnan@med.ovgu.de (S.K.); rajiv.rana@medizin.uni-leipzig.de (R.R.); hamzah.khawaja@medizin.uni-leipzig.de (H.K.); dheerendra.gupta@medizin.uni-leipzig.de (D.G.); jayakumar.manoharan@medizin.uni-leipzig.de (J.M.); shrey.kohli@medizin.uni-leipzig.de (S.K.); berend.isermann@medizin.uni-leipzig.de (B.I.); 2 Institute of Experimental Internal Medicine, Medical Faculty, Otto von Guericke University, 39120 Magdeburg, Germany; 3 Cardiology, Leipzig Heart Center, University of Leipzig, 04289 Leipzig, Germany; christian.besler@medizin.uni-leipzig.de; 4 Klinik und Poliklinik für Kardiologie, University Hospital Leipzig, 04103 Leipzig, Germany; ulrich.laufs@medizin.uni-leipzig.de

**Keywords:** atherosclerosis, diabetes, senescence, unfolded protein response, endothelial cells, activated protein C

## Abstract

Diabetes mellitus is hallmarked by accelerated atherosclerosis, a major cause of mortality among patients with diabetes. Efficient therapies for diabetes-associated atherosclerosis are absent. Accelerated atherosclerosis in diabetic patients is associated with reduced endothelial thrombomodulin (TM) expression and impaired activated protein C (aPC) generation. Here, we directly compared the effects of high glucose and oxidized LDL, revealing that high glucose induced more pronounced responses in regard to maladaptive unfolded protein response (UPR), senescence, and vascular endothelial cell barrier disruption. Ex vivo, diabetic ApoE^−/−^ mice displayed increased levels of senescence and UPR markers within atherosclerotic lesions compared with nondiabetic ApoE^−/−^ mice. Activated protein C pretreatment maintained barrier permeability and prevented glucose-induced expression of senescence and UPR markers in vitro. These data suggest that high glucose-induced maladaptive UPR and associated senescence promote vascular endothelial cell dysfunction, which—however—can be reversed by aPC. Taken together, current data suggest that reversal of glucose-induced vascular endothelial cell dysfunction is feasible.

## 1. Introduction

Cardiovascular disease (CVD) is the leading pathology associated with diabetes mellitus. The unprecedented increase of diabetes and disproportionately higher CVD mortality in patients with diabetes compared to nondiabetic patients are both alarming [1,2]. The risk of atherosclerotic is greater in both type 1 and type 2 diabetic patients than non-diabetic patients. Moreover, patients with type 1 or type 2 diabetes display earlier onset and more advanced atherosclerotic plaques in comparison with age-matched patients without diabetes [3,4]. Plaque morphology differs among diabetic and non-diabetic atherosclerotic disease, suggesting at least partially different disease pathology.

Diabetes-associated atherosclerosis is characterized by a larger necrotic core area, thin-cap atheroma, and a more pronounced inflammatory cell infiltrate independent of other risk factors [5]. Stable plaques are characterized by a relatively thicker fibrous cap and higher vascular smooth muscle cells (VSMCs) content.

Diabetes aggravates atherosclerosis progression and impairs plaque regression [6,7]. Reduction in atherosclerotic plaques after aggressive lipid lowering has been shown in animal and clinical studies [8]. Intriguingly, atherosclerosis regression following lipid lowering is impaired in both humans [9] and mice [6,7] with diabetes. Furthermore, clinical trials evaluating statins revealed that cardiovascular events are more frequent in patients with diabetes than patients without diabetes [10]. However, diabetes-specific mechanisms resulting in diabetes-associated atherosclerosis remain largely unknown, and we lack specific therapeutic approaches.

Reduced activated protein C (aPC) generation is linked with atherosclerosis [11,12] and diabetes mellitus [13,14]. Likewise, protein expression of thrombomodulin (TM) and the endothelial protein C receptor (EPCR) is decreased in endothelial cells of atherosclerotic coronary arteries [15]. In our previous studies in mice, impairment of atherosclerosis regression was assigned in part to hyperglycaemia-induced sustained production of oxidative stress, proinflammatory cytokines, and lipid uptake by macrophages [16]. Notably, plaque macrophages in diabetic mice persistently showed signs of increased inflammation and reactive oxygen species (ROS) levels despite glucose lowering [16]. Next, in the case of macrophage dysfunction, endothelial dysfunction is an important contributor in the pathobiology of atherosclerosis [17]. While several studies have shown that risk factors such as hyperglycaemia, associated advance glycation end products (AGEs), and high cholesterol levels contribute to endothelial dysfunction, studies directly comparing the impact of hyperglycaemia versus hyperlipidaemia on endothelial dysfunction are lacking. Mechanistically, unfolded protein response (UPR) and vascular senescence have been observed in atherosclerosis; however, the relative contribution of hyperglycaemia versus hyperlipidaemia to maladaptive UPR and associated vascular senescence in diabetes-accelerated atherosclerosis has noy hitherto been studied.

The endoplasmic reticulum (ER) responds to stress insults by activating intracellular signaling pathways, named as the unfolded protein response (UPR) [18,19]. Activated transcription factor (ATF6) and spliced X-box binding protein (sXBP1) are two important UPR signal transducer proteins [18,19]. Both ATF6 and sXBP1 are closely linked with diabetes and atherosclerosis [20]. The aim of the current study was to characterize the molecular alterations of a dysfunctional vascular endothelium in response to hyperglycaemic versus hyperlipidaemic conditions. We hypothesize that under hyperglycaemic versus hyperlipidaemic conditions, endothelium will display distinct molecular and functional differences. The identification of such differences will provide new insights on the pathomechanisms triggering endothelial dysfunction in diabetes-accelerated atherosclerosis.

## 2. Materials and Methods:

### 2.1. Reagents

The following reagents and antibodies were used in this study: p21 Waf1/Cip1 (12D1) rabbit, p16 INK4A (D7C1M) rabbit, p53 (1C12) mouse (Cell Signaling Technology, Frankfurt, Germany); anti-phospho-IRE1 (S724) rabbit monoclonal antibody (Boster, Germany); human XBP1 antibody (R & D System, Wiesbaden, Germany); anti ATF6 alpha (Rockland, Germany); alexa fluor FITC goat anti-rabbit IgG, alexa fluor TRITC goat anti-rabbit IgG, alexa fluor TRITC goat anti-mouse IgG and alexa fluor FITC goat anti-mouse IgG (Invitrogen, Karlsruhe, Germany). Human coronary artery endothelial cells were purchased from Lonza Germany (Catalog #: CC-2585); EGMTM -2 MV microvascular endothelial cell growth medium-2 bulletkit (Lonza, Köln, Germany); D-(+)-Glucose (Sigma, Darmstadt, Germany); human oxidized low density lipoprotein (oxLDL) (Thermo Fisher Scientific, Dreieich, Germany); vectashield mounting medium with DAPI (Vector Laboratories, Newark, CA, USA); streptozotocin (Enzo Life Sciences, Lörrach, Germany); “high-fat diet” (HFD) experimental food (Western Type Diet, 43% carbohydrates, 15% proteins and 42%, Ssniff, Soest, Germany); accu-chek test strips, accu-check glucometer; albumin fraction V (Carl Roth, Karlsruhe, Germany); rompun 2% (Bayer, Leverkusen, Germany); ketamine 10% (beta-pharm, Augsburg, Germany); chemiluminescent western blot reagents (Merk Millipore, Darmstadt, Germany).

### 2.2. Mice

ApoE^−/−^ (002052) mice were obtained from the Jackson Laboratory (Bar Harbor, ME, USA). In the current study we used littermates backcrossed for at least 10 generations on a C57BL/6J background. Only age-matched mice were used throughout the study. All animal experiments were conducted following standards and procedures approved by the local Animal Care and Use Committee ((IKCP/G/05-1083/11_Maus, 30.01.2010, Landesverwaltungsamt, Halle, Germany).

### 2.3. Atherosclerotic Mouse Models

Female ApoE^−/−^ mice (age 6 to 8 weeks) were fed a high-fat diet (HFD) or normal chow diet, and were made diabetic (DM) by injecting streptozotocin (STZ, 60 mg/kg, intraperitoneally, once daily for five consecutive days, freshly dissolved in 0.05 M sterile sodium citrate, pH 4.5), reflecting type 1 DM [12,16,21,22,23]. Control mice were injected with an equal volume of 0.05 M sodium citrate, pH 4.5 for 5 days. Blood glucose and body weight were measured once weekly [12,23]. On average, 85–90% of mice became diabetic (blood glucose > 300 mg/dL) within the first 4 weeks, and these were included as diabetic mice in the experiments. Mice not developing persistently elevated blood glucose levels and maintaining blood glucose levels < 200 mg/dl despite STZ-injections were included in the control group [12,23]. The endpoints analysed did not differ between HFD fed mice, STZ-injected, but normoglycemic mice and sodium-citrated injected mice. HFD or hyperglycaemia (minimum 300 mg/dL) was maintained for up to 22 weeks.

### 2.4. Cell Culture

Human coronary artery endothelial cells (HCAECs) were commercially obtained (Lonza, Köln, Germany). These HCAECs were routinely grown and maintained in endothelial cell-low glucose (5.5 mM glucose concentration) growth medium in the presence of 10% FBS at 37 °C, according to manufacturer’s instructions.

### 2.5. Immunofluorescence Staining

For immunofluorescence staining on cells, HCAECs were cultured on cover slips and challenged with either normal (5 mM), high glucose (25 mM) or oxidized low density lipoprotein (50 µg/mL) with or without exogenous wild type aPC (20 nM). For immunofluorescence, frozen sections of brachiocephalic arteries with maximum plaque size were used. Tissue sections or cells were fixed in ice cold acetone for 8 min, washed twice with ice cold PBS, and incubated in 2% BSA in PBST for 1 h. Tissue sections or cells were then incubated overnight at 4 °C with primary antibodies against p21, p16, p53 (1:200), sXBP1 (1:50), and ATF6 alpha (1:50). Sections or cells incubated without primary antibodies were used as negative controls for background correction. After overnight incubation, the sections were washed three times with 1 × PBS for 5 minutes each time, followed by incubation with fluorescently labelled corresponding secondary antibodies. After washing, nuclear counterstaining was conducted using mounting medium with DAPI. Images were visualized, captured, and analysed using a fluorescence microscope. All histological analyses were performed by two independent blinded investigators. Immunofluorescence images were captured with an Olympus Bx43-Microscope (Olympus, Hamburg, Germany). Image J software was used for image analysis.

### 2.6. Senescence-Associated Beta Galactosidase Staining

Endothelial cell senescence was analysed using senescence cells staining kit (CS0030-Sigma) following the manufacturer’s protocol. Briefly, HCAECs were cultured on cover slips and exposed to either normal (5 mM), high glucose (25 mM) or oxidized low density lipoprotein (50 µg/mL). Cells were washed with ice cold 1 × PBS and fixed for 1 min (2% formaldehydes, 0.2% glutaraldehyde, 7.04 mM Na2HPO4, 1.47 mM KH2PO4, 0.137 M NaCl, 2.68 mM KCl) and then rinsed with ice-cold PBS for 5 min. Cells were incubated with freshly prepared X-gal staining solution (10 mL containing 0.25 mL of 40 mg/mL X-gal solution, 125 µL of 400 mM potassium ferricyanide, 125 µL of 400 mM potassium ferrocyanide) at 37 °C overnight. Next day, cover slips were washed with ice cold PBS to remove excess staining solution. Cover slips were covered with 70% glycerol and mounted. Images were visualized, captured, and analysed using Olympus Bx43-Microscope (Olympus, Hamburg, Germany).

### 2.7. Immunoblotting

Cell lysates were prepared in RIPA buffer (50 mM Tris at pH 7.4, 1% Nonidet P-40, 0.25% sodium deoxycholate, 150 mM NaCl, 1 mM EDTA, 1 mM Na3VO4, and 1 mM NaF supplemented with a protease inhibitor cocktail). Lysates were centrifuged (10.000× *g*, 10 min at 4 °C) and insoluble debris was discarded. The protein concentration in supernatants was quantified using BCA reagent. Equal amounts of protein were electrophoretically separated on 7.5%, 10% (*v*/*v*) or 12.5% (*v*/*v*) SDS polyacrylamide gels, as appropriate, transferred to PVDF membranes, and probed with the desired primary antibodies overnight at 4 °C. Membranes were then washed with PBS-Tween (PBST) and incubated with anti-mouse IgG, anti-rat IgG or anti-rabbit IgG (each 1:4000) horseradish peroxidase-conjugated antibodies, as indicated. Blots were developed with an enhanced chemiluminescence system. To compare and quantify the levels of proteins, the density of each band was measured using Image J. Equal loading was confirmed by immunoblotting with a GAPDH antibody.

### 2.8. Fluorescein Isothiocyanate (FITC)-Dextran Vascular Endothelial Cells Permeability Assay

FITC dextran permeability assay was conducted to measure the integrity of vascular endothelial barrier in vitro. The model consists of a polyethyleneterephthalate (PET) membrane (Millipore membrane, Millicell Hanging Cell Culture Insert) with 1 µm pores, coated with collagen type IV. This model enables functional permeability assays through fluorometric measurements of the transmembrane passage of FITC-labelled dextran. Briefly, HCAECs were grown on the membrane-side, facing upwards until confluency. The cell monolayer was exposed to either normal (5 mM), high glucose (25 mM) or oxidized low density lipoprotein (50 µg/mL) with or without exogenous wild type aPC (20 nM) for 48 h. FITC dextran was added on the upper chamber of the transwell, and after 1 h, barrier permeability was assessed by measuring the passage of FITC dextran across the transwell in the lower chamber. The fluorescence was measured using fluorometric spectrophotometer at 495 nm and the concentration of FITC dextran was calculated by reference to a set of standard dilutions. For the percent barrier integrity measurement, first % barrier disruption was calculated using following formula:

% barrier disruption = FITC dextran measured in the lower transwell chamber in the presence of cells/FITC dextran measured in the lower transwell chamber in the absence of cells × 100

% barrier integrity = 100 − % barrier disruption

Cells exposed to mannitol (20 mM) plus normal glucose concentration (5.5 mM glucose) and normal lipids served as controls.

### 2.9. Transendothelial Electrical Resistance (TEER) Assay

Transepithelial/transendothelial electrical resistance (TEER) assay was conducted to measure the integrity of vascular endothelial barrier in vitro. HCAECs were seeded on transwell (Millicell hanging cell culture insert) and maintained at 37 °C with 5% CO_2_. After reaching confluency/monolayer, cells were exposed to either normal glucose, high glucose, or oxLDL with or without aPC as described above (under section: Fluorescein isothiocyanate (FITC)-dextran vascular endothelial cells permeability assay). Barrier integrity was determined using a TEER machine. Briefly, the TEER meter probe was placed into each well insert (short probe in transwell (apical side), the long probe into the receiver well (basolateral side)) and electrical resistance (in ohm) was recorded. TEER was calculated as follows; the surface area of the transwell (in cm^2^) was multiplied by the net electrical resistance (which is the resistance measured minus the resistance of a blank transwell covered by cell culture media only).

### 2.10. Statistical Analysis

Female mice were grouped according to genotype and randomly assigned to different interventions (control, streptozotocin, high fat diet). The data are summarized as mean ± standard error of the mean (SEM). Statistical analyses were performed with Mann–Whitney U test or analysis of variance (ANOVA) as appropriate. Post hoc comparisons of ANOVA were corrected with the method of Bonferroni. The Kolmogorov–Smirnov test or D’Agostino–Pearson normality test was used to determine whether the data are consistent with Gaussian distribution. Prism 8 (www.graphpad.com, accessed on 30 April 2022) software was used for statistical analyses. Values of *p* ≤ 0.05 were considered statistically significant.

## 3. Results

### 3.1. High Glucose-Induced Senescence Promotes Barrier Disruption in Vascular Endothelial Cells

**Figure 1:**
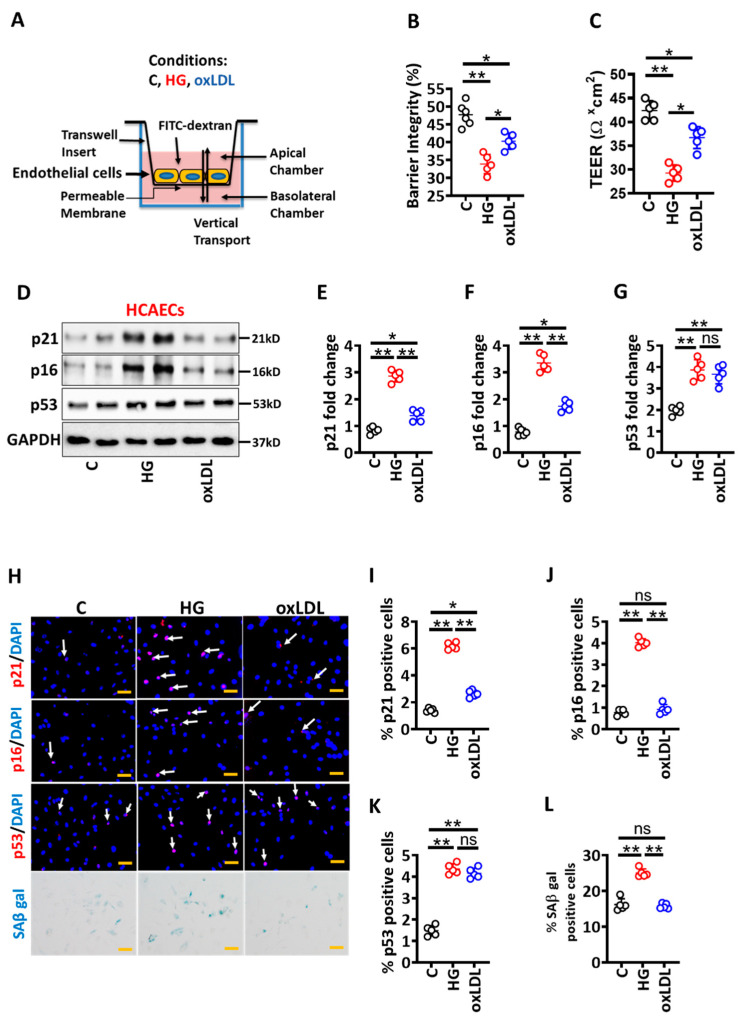
Hyperglycaemia-induced senescence promotes barrier disruption in endothelial cells. (**A**) Experimental design and treatment conditions. (**B**,**C**) Dot plot summarizing data of barrier integrity measured by FITC dextran leakage (**B**) and TEER (**C**). (**D**–**G**) Exemplary immunoblot of p21, p16, and p53 ((**D**), loading control: GAPDH) and dot plot summarizing p21 (**E**), p16 (**F**) and p53 (**G**) densitometric quantifications of immunoblots. (**H**–**L**) Representative immunofluorescence images showing endothelial cell staining for p21 ((**H**), top panel, red; nuclear counterstain: DAPI, blue), p16 ((**H**), first middle panel, red; nuclear counterstain: DAPI, blue), p53 ((**H**), second middle panel, red; nuclear counterstain: DAPI, blue) and senescence-associated beta galactosidase staining ((**H**), bottom panel, blue). Dot plots summarizing data for p21 (**I**), p16 (**J**), p53 (**K**) and SAβ gal (**L**). Scale bar: 20 µm (**H**). HCAEC maintained under control (non-treated, C), high glucose (25 mM; HG) or oxidized low density lipoprotein (oxLDL 50 µg/mL, oxLDL) conditions. Each dot represents data obtained from one biological specimen; * *p* < 0.05, ** *p* < 0.01; ANOVA.

To gain insights into specific pathomechanisms of hyperglycaemia versus hyperlipidaemia-induced vascular endothelial cells dysfunction, we directly compared the impact of glucose versus oxLDL on human coronary artery endothelial cells (HCAECs). HCAECs were cultured under normal conditions (C), high glucose (25 mM glucose, HG) or oxidized low density lipoprotein (oxLDL, 50 µg/mL) conditions for 48 h (Figure 1A). The oxLDL modestly increased endothelial barrier permeability (indicated by increased FITC-dextran leakage and reduced TEER), while HG (but not control, C; Figure 1B,C) markedly impaired endothelial barrier permeability (Figure 1B,C). Thus, HG-mediated impairment of vascular endothelial barrier is more pronounced than oxLDL, suggesting that hyperglycaemia-induced endothelial dysfunction contributes to accelerated atherosclerosis in diabetes mellitus.

To identify pathomechanisms underlying differential effects of HG versus oxLDL on the barrier integrity of vascular endothelial cells, we determined protein levels of senescence markers (p21, p16 and p53) under the above conditions. Given the critical pathogenic role of senescence at all stages of atherosclerosis development [24,25], we considered that senescence may be a potential mechanism by which HG and oxLDL exert differential effects in atherosclerosis. While both oxLDL and HG induced p53 levels to a similar extent, HG but not oxLDL markedly induced these markers in HCAECs (Figure 1D–G), corroborating the differential impact of oxLDL versus HG on vascular endothelial cells senescence. Consistent with immunoblotting data, immunofluorescence analyses revealed increased p21 and p16 expression in HG-exposed HCAECs (Figure 1H–K). To ascertain the role of senescence, we next performed senescence-associated β-galactosidase (SA-β-gal) staining. SA-β-gal staining was markedly increased in HG-induced HCAECs (Figure 1H–L). Induction of senescence in vascular endothelial cells has been reported, but the relative contribution of HG versus oxLDL has not hitherto been shown. Thus, hyperglycaemia-induced senescence is associated with impaired barrier integrity.

**Figure 2:**
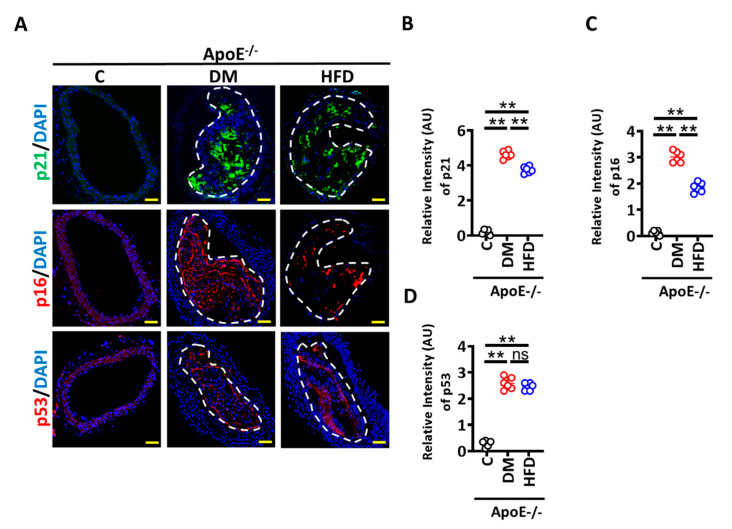
Senescence is induced within plaques of diabetic ApoE^−/−^ mice. (**A**) Representative immunofluorescence images showing staining of brachiocephalic arteries for p21 ((**A**), top panel, p21, green; nuclear counterstain: DAPI, blue), p16 ((**A**), middle panel, p16, red; nuclear counterstain: DAPI, blue) and p53 ((**A**), bottom panel, p16, red; nuclear counterstain: DAPI, blue). (**B**–**D**) Dot plots summarizing immunofluorescence data for p21 (**B**) p16 (**C**), p53 (**D**) and Scale bar: 20 µm (**A**). ApoE^−/−^ control mice (Cont, normal chow diet, citrate instead of streptozotocin injections), DM mice (normal chow diet, streptozotocin injections) or HFD mice (fed high fat diet). Each dot represents data obtained from one mouse specimen; ** *p* < 0.01; ANOVA.

To find the in vivo relevance of these findings, we determined p21, p16, and p53 levels in the brachiocephalic artery of ApoE^−/−^ mice with persistent hyperglycaemia (induced by low-dose streptozotocin, STZ injection for 5 days, a model of type 1 DM) or hyperlipidaemia (induced by a high-fat diet, HFD) for 22 weeks [16]. Diabetic ApoE^−/−^ mice display smaller plaques with thinner fibrous caps as compared with HFD ApoE^−/−^ mice (Supplementary Figure S1) [16]. Within the study period, only small plaques were observed in a subset of ApoE^−/−^ on a normal chow diet, precluding a histological analysis. Immunohistochemical analyses verified an increased expression of p21 and p16 within plaques of diabetic ApoE^−/−^ versus HFD ApoE^−/−^ mice (Figure 2A–C). Consistent with in vitro data, p53 was induced to similar levels in diabetic ApoE^−/−^ versus HFD ApoE^−/−^ mice (Figure 2A,D). Thus, atherosclerotic plaques of diabetic ApoE^−/−^ mice display more p21 and p16 compared to non-diabetic mice.

### 3.2. High Glucose Mediated Maladaptive UPR Signalling Impairs Vascular Endothelial Barrier Integrity

**Figure 3:**
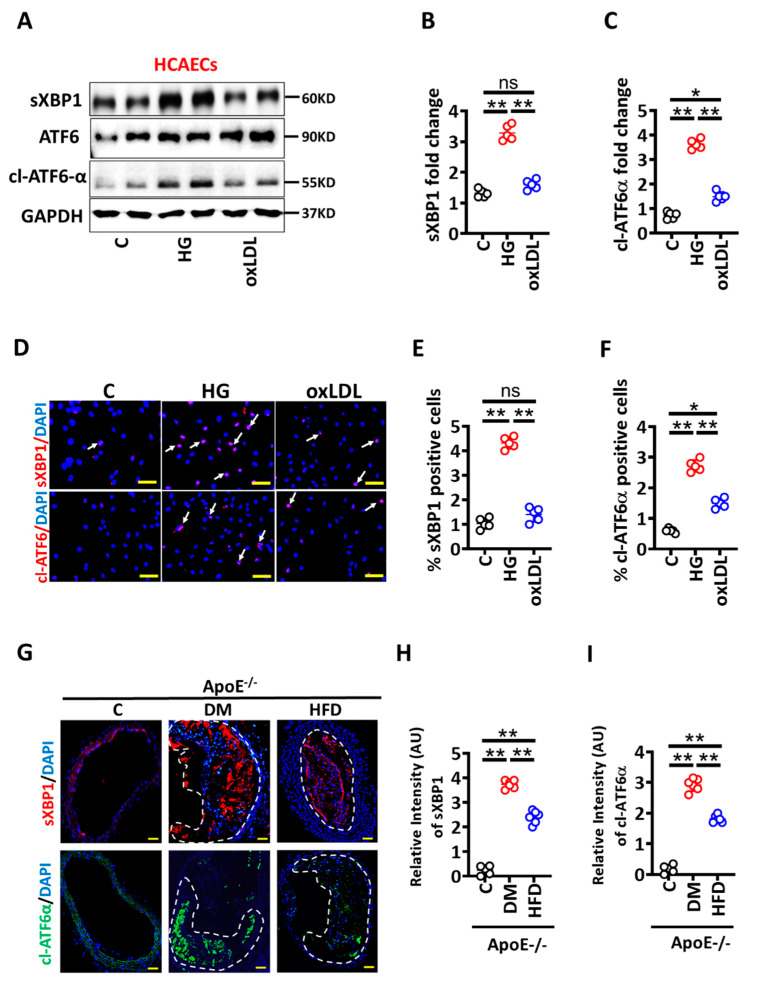
Hyperglycaemia induces UPR in endothelial cells and within plaques of diabetic ApoE^−/−^ mice. (**A**–**C**) Representative immunoblots spliced XBP1 (sXBP1), total ATF6, and cleaved ATF6 alpha (cl-ATF6α) ((**A**), loading control: GAPDH). Dot plots summarize densitometric quantifications of immunoblotting results for sXBP1 (**B**) and cl-ATF6α (**C**). (**D**–**F**) Representative immunofluorescence images showing endothelial cells staining for sXBP1 ((**D**), upper panel, red; nuclear counterstain: DAPI, blue) and cl-ATF6-α ((**D**), lower panel, red; nuclear counterstain: DAPI, blue). Dot plots summarizing immunofluorescence data for sXBP1 (**E**) and cl-ATF6-α (**F**). Scale bar: 20 µm (**D**). (**G**–**I**) Representative immunofluorescence images showing staining of brachiocephalic arteries for sXBP1 ((**G**), upper panel, sXBP1, red; DAPI nuclear counterstain, blue) and cl-ATF6-α ((**G**), lower panel, cl-ATF6-α, green; DAPI nuclear counterstain, blue). Dot plots summarizing immunofluorescence data for sXBP1 (**H**) and cl-ATF6-α (**I**). Scale bar: 20µm (**G**). HCAECs maintained under control (**C**), high glucose (25 mM; HG) or oxidized low density lipoprotein (oxLDL 50 µg/mL, oxLDL) conditions. Each dot represents data obtained from one biological specimen. ApoE^−/−^ control mice (Cont, normal chow diet, citrate instead of streptozotocin injections), DM mice (normal chow diet, streptozotocin injections) or HFD mice (fed high fat diet). Each dot represents data obtained from one mouse specimen; * *p* < 0.05, ** *p* < 0.01, ns: non-significant; ANOVA.

We next investigated the mechanism regulating enhanced senescence induction in HG conditions. p53 was induced to the same extent in HG or oxLDL conditions in HCAECs, suggesting that p53-independent pathways contribute to hyperglycaemia-induced senescence and barrier dysfunction. Senescence has been mechanistically linked to the maladaptive unfolded protein response (UPR) [26,27], and as both are linked with atherosclerosis [20,24], we next determined whether maladaptive UPR contributes to HG-induced senescence and impaired barrier integrity. To this end, we determined markers of UPR, sXBP1 (spliced X-box binding protein), and cl-ATF6 α (cleaved, activating transcription factor 6 alpha) [18,19] in HG versus oxLDL exposed HCAECs in vitro. Immunoblotting analysis revealed markedly induced sXBP1 and cl-ATF6 α levels in HG versus oxLDL exposed HCAECs (Figure 3A–C). Consistent with these data, immunofluorescence staining verified increased sXBP1 and cl-ATF6α levels in HG versus oxLDL exposed HCAECs (Figure 3D–F).

To determine the in vivo relevance of these results, we evaluated sXBP1 and cl-ATF6 α levels in the brachiocephalic artery of ApoE^−/−^ mice with persistent hyperglycaemia or hyperlipidaemia [16]. Immunohistochemical analyses demonstrated increased expression of sXBP1 and cl-ATF6α within plaques of DM ApoE^−/−^ versus HFD ApoE^−/−^ mice (Figure 3G–I). Thus, atherosclerotic plaques of hyperglycaemic mice express more sXBP1 and cl-ATF6α compared to hyperlipidaemic mice. Taken together these data suggest that hyperglycaemia-associated impaired vascular integrity primarily depends on maladaptive UPR and senescence pathways.

### 3.3. Activated Protein C Restricts the UPR and Prevents High Glucose-Induced Senescence in HCAECs

**Figure 4:**
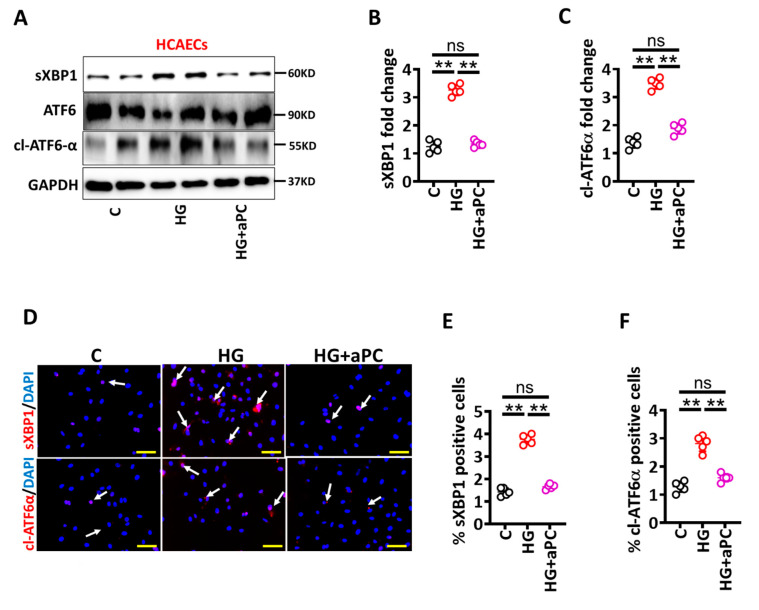
aPC avert glucose-induced UPR. (**A**–**C**) Representative immunoblots showing spliced XBP1 (sXBP1), total ATF6 and cleaved ATF6 alpha (cl-ATF6α) ((**A**), loading control: GAPDH). Dot plots summarize densitometric quantifications of immunoblotting results for sXBP1 (**B**) and cl-ATF6α (**C**). (**D**–**F**) Representative immunofluorescence images showing staining for sXBP1 ((**D**), middle panel, red; nuclear counterstain: DAPI, blue) and cl-ATF6-α ((**D**), bottom panel, red; nuclear counterstain: DAPI, blue). Dot plots summarizing immunofluorescence data for sXBP1 (**E**) and cl-ATF6-α (**F**). Scale bar: 20 µm (**D**). HCAECs maintained under control (**C**), high glucose (25 mM; HG) or HG + aPC (25 mM glucose + 20 nM of exogenous activated protein C) conditions. Each dot represents data obtained from one biological specimen. ** *p* < 0.01; ANOVA.

**Figure 5:**
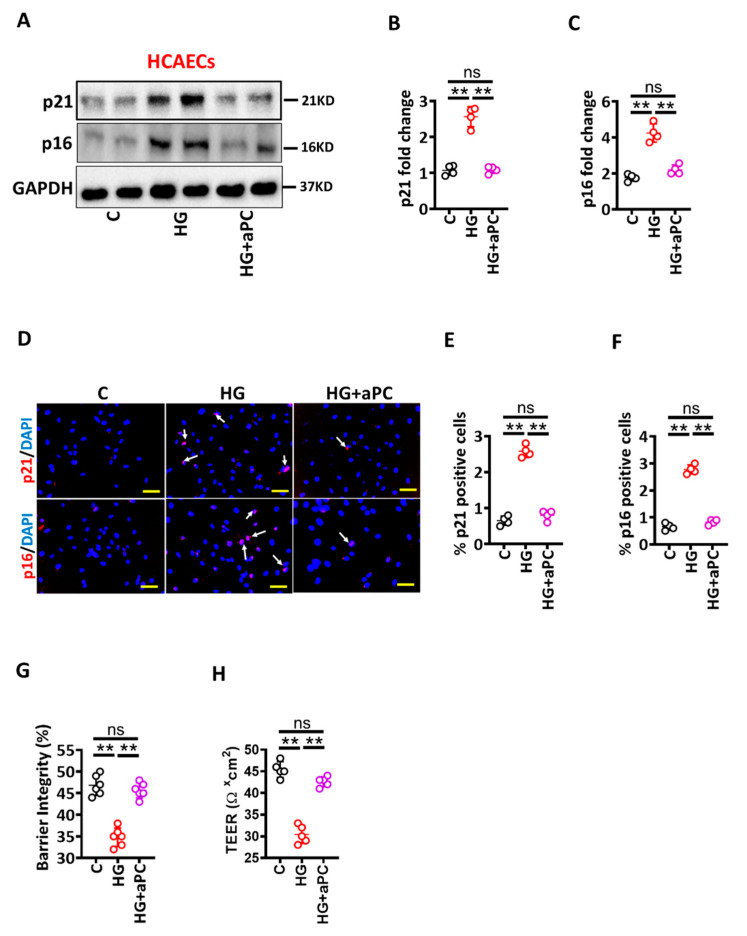
aPC reduces glucose-induced senescence. (**A**–**C**) Representative immunoblots showing p21 and p16 expressions ((**A**), loading control: GAPDH). Dot plots summarize densitometric quantifications of immunoblotting results for p21 (**B**) and p16 (**C**). (**D**–**F**) Representative immunofluorescence images showing endothelial cells staining for p21 ((**D**), upper panel, p21, red; nuclear counterstain: DAPI, blue) and p16 ((**D**), lower panel, p16, red; nuclear counterstain: DAPI, blue). Dot plots summarizing immunofluorescence data for p21 (**E**) and p16 (**F**). Scale bar: 20 µm (**D**). (**G**,**H**) Dot plot summarizing barrier integrity data measured by FITC dextran leakage (**G**) and TEER (**H**). HCAECs maintained under control (**C**), high glucose (25 mM; HG), or HG+aPC (25 mM glucose + 20nM of exogenous activated protein C) conditions. Each dot represents data obtained from one biological specimen. ** *p* < 0.01; ANOVA.

Hyperglycaemia is linked with endothelial dysfunction and diminished thrombomodulin (TM)-dependent protein C activation (aPC) [13], and aPC has been shown to restrict maladaptive UPR-signaling [12]. To determine whether pre-treatment of exogenous aPC prevents glucose-induced senescence in HCAECs, we analysed UPR markers in HCAECs cultured under control and high glucose conditions. Immunoblotting analysis revealed that HG-induced UPR markers (sXBP1 and cl-ATF6 α) were prevented by aPC pre-treatment (HG + aPC, Figure 4A–C). Analysis of immunofluorescence stained images validated immunoblotting data (Figure 4D–F). Thus, aPC prevents HG-induced maladaptive UPR induction in HCAECs. We next determined levels of senescence markers and barrier permeability under the above conditions. Consistent with UPR data, aPC pre-treatment abolished HG-induced p21 and p16 expressions and improved barrier integrity in HCAECs (Figure 5).

Taken together, these data suggest that HG-induced ER stress drives vascular endothelial cells dysfunctions in the context of diabetes-accelerated atherosclerosis.

## 4. Discussion

Cardiovascular complications such as myocardial infarction (also called a heart attack), cerebrovascular events, and peripheral vascular complications are the major cause of death among diabetic patients [11]. Decreased thrombomodulin expression in vascular endothelium and plasma aPC levels have been linked with atherosclerosis- and diabetes-associated vascular complications [13,14,15,21,28]. Here, we demonstrate that HG (as compared to oxLDL) induced pronounced maladaptive unfolded protein response (UPR) and senescence which promotes vascular endothelial cell dysfunction, as reflected by impaired endothelial cell barrier. Activated protein C restored barrier integrity, reduced expression of senescence and UPR markers in glucose-exposed vascular endothelial cells in vitro. Ex vivo, diabetic ApoE^−/−^ mice displayed increased levels of senescence and UPR markers within atherosclerotic plaque as compared with nondiabetic ApoE^−/−^ mice on an HFD. The current data suggest that targeting maladaptive UPR using aPC-based approaches may help to prevent vascular dysfunction in diabetes-accelerated atherosclerosis.

By directly comparing HG versus oxLDL conditions in vitro, we first show that HG and oxLDL differentially impair vascular endothelial barrier disruption. oxLDL-induced barrier impairment was less pronounced than HG-induced barrier disruption. Although previous studies have shown high lipid and high glucose-induced vascular endothelial cells barrier disruption individually [29,30,31], these studies did not directly compare the effect of oxLDL versus HG conditions on vascular endothelial cells barrier disruption. Future in vivo studies are needed to determine the functional relevance of UPR signaling in endothelial cells for atherogenesis in diabetic versus hyperlipidemic mice and patients.

We next show that senescence is associated with endothelial dysfunction in HG conditions. While oxLDL also induced markers of senescence and UPR compared to control, HG more profoundly inducted senescence and UPR markers. Furthermore, increased levels of senescence and UPR markers were detectable within the atherosclerotic plaques of hyperglycaemic ApoE^−/−^ mice as compared to hyperlipidaemic ApoE^−/−^ mice. These findings add to previous studies, demonstrating that development of atherosclerosis is closely linked with senescent cells at all stages of atherosclerosis [24]. Moreover, senescent cells are known to contribute to plaque instability, including degradation of elastic fiber and fibrous cap thinning [24]. As atherosclerotic plaques in diabetic settings display signs of instability (for example, thinner fibrous cap) [5,16,32,33,34] and increased levels of senescence markers were detected in atherosclerotic plaques of diabetic mice in the current study, we hypothesize that hyperglycaemia-induced senescence may promote accelerated atherosclerosis and plaque instability in diabetic patients; however, this needs to be evaluated in future studies. Notably, atherosclerotic plaque morphology in diabetic ApoE^−/−^ mice, characterized by hyperglycaemia and hyperlipidaemia, mimics the situation observed frequently in cardiac patients with diabetes mellitus. Given the current results, we cannot, however, exclude that other diabetes-associated factors, such as obesity or impaired insulin signaling, contribute to a plaque instability in diabetic patients.

Here, maladaptive UPR was identified as one pathway modulating expression of senescence markers under diabetes conditions. UPR has been linked with senescence before [26,27]. Intriguingly, senescence is considered as a stress response that arrests altered cells following UPR [26]. While we demonstrate that increased cl-ATF6 α and sXBP-1 levels are critical for diabetes-induced senescence within plaques of diabetic mice and, in hyperglycaemia-exposed endothelial cells, the PERK/ATF4 arm was not studied. Of note, the three “canonical” branches of UPR are not always activated together in the context of senescence, and are not specific to a certain type of senescence. Whether all three arms of UPR levels contribute to diabetes-induced senescence and endothelial dysfunction needs to be evaluated in future studies. Evaluation of the complex modulating the UPR-linked senescence may identify additional therapeutic targets. This may aid the formulation of better treatment approaches in the context of diabetes-associated atherosclerosis.

Here, we demonstrate that the coagulation protease aPC prevents UPR- and senescence-induced endothelial dysfunction in diabetes conditions. These data are consistent with our previous work demonstrating that aPC prevents maladaptive UPR in podocytes in diabetic conditions [12] and conveys anti-atherogenic effects in macrophages under diabetic conditions [16]. Whether the regulation of UPR in endothelial cells and mitochondrial ROS in macrophages by aPC in the context of diabetes are mechanistically linked requires future work. The regulation of UPR and senescence by aPC suggest that aPC may prevent high glucose-induced endothelial dysfunction in diabetes-accelerated atherosclerosis. Furthermore, aPC has shown benefits in other acute and chronic disease models [13,16,35,36]. aPC mutants lacking anticoagulation properties (3K3A-aPC) or small molecules mimicking biased signaling of aPC (parmdoulin) [37] make aPC-based therapies suitable for both acute and also chronic diseases [13,16,35,36].

While providing novel insights, the current study has potential limitations. We intended to directly compare the impact of oxLDL versus HG on endothelial dysfunction. Based on available data, we focused on UPR and senescence, but cannot exclude that unbiased approaches (e.g., RNAseq) may identify other molecular pathways activated in oxLDL versus HG conditions. However, we are confident that the observed glucose-induced changes are relevant, as the endpoints analysed (e.g., UPR and senescence) have been linked with atherogenesis before [24,25,38]. Moreover, due to restriction in the permission for in vivo mouse work and following the 3R (Replacement, Reduction and Refinement) principle, we could only include female mice in the current study. Considering the gender-dependent differences in atherosclerosis development, future studies using female and male mice would help to better understand gender-specific pathomechanisms. Furthermore, in the current study, we studied a mouse model of type 1 diabetes mellitus. Although the streptozotocin-induced type 1 diabetic model is an established model and is considered to be suitable for investigating diabetes-associated atherosclerosis, we cannot exclude the impact of metabolic changes observed in type 2 diabetic patients, such as obesity or insulin resistance, on plaque stability.

Despite these potential limitations, the current study shows that aPC targets UPR and senescence and maintains endothelial function in high glucose conditions. Targeting UPR using chaperone or using aPC or aPC-mimetics such as parmodulin may allow the specific combatting of accelerated atherosclerosis in diabetes mellitus.

## 5. Conclusions

The main cause of mortality in diabetic patients is atherosclerosis and its associated complications. While the more aggressive disease progression of atherosclerosis in diabetic patients is established, the underlying mechanisms remain obscure, hampering specific therapeutic approaches to atherosclerosis in diabetic patients. Here, we directly compared the impact of glucose versus lipid on endothelial cells and provide new insights into glucose- versus lipid-induced endothelial dysfunction. We establish that glucose more than oxLDL causes endothelial barrier impairment, which is associated with an enhanced unfolded protein response and cellular senescence. In parallel, levels of UPR and senescence markers were higher in diabetic atherosclerotic lesions, but not in mice without diabetes mellitus. The cytoprotective protease aPC prevents glucose-induced UPR and senescence in endothelial cells. These findings suggest that hyperglycaemia-induced maladaptive UPR and associated senescence may represent a pathomechanism contributing to differential effects of glucose versus oxLDL on endothelial dysfunction and atherosclerosis. These insights advance our understanding of the pathomechanism underlying diabetes-accelerated atherosclerosis.

## Supplementary Materials

The following supporting information can be downloaded at: https://www.mdpi.com/article/10.3390/nu14142786/s1, Figure S1: Smaller, but less stable plaques in hyperglycaemic versus hyperlipidaemic ApoE-deficient mice.

## Abbreviations

aPC	activated protein C
ApoE	apolipoprotein E
ASCVD	atherosclerotic cardiovascular disease
ANOVA	analysis of variance
DM	diabetes mellitus
DAPI	4′,6-diamidino-2-phenylindole
DEPC	diethyl pyrocarbonate
EPCR	endothelial protein C receptor
NF-κB	nuclear factor kappa-light-chain-enhancer of activated B cells
qPCR	quantitative polymerase chain reaction
ROS	reactive oxygen species
RT-PCR	reverse transcriptase polymerase chain reaction
SMC	smooth muscle cells
α-SMC	alpha-smooth muscle actin
SEM	standard error mean
STZ	streptozotocin
TM	thrombomodulin
