# Supplementary material for: ER-Stress and Senescence Coordinately Promote Endothelial Barrier Dysfunction in Diabetes-Induced Atherosclerosis
Source: Nutrients. 2022 Jul 6;14(14):2786. doi: 10.3390/nu14142786 (PMC9323824; doi:10.3390/nu14142786)
Supplement: Supplementary file 1 [file nutrients-14-02786-s001.zip › nutrients-1768092-supplementary.pdf]

Supplementary Figure

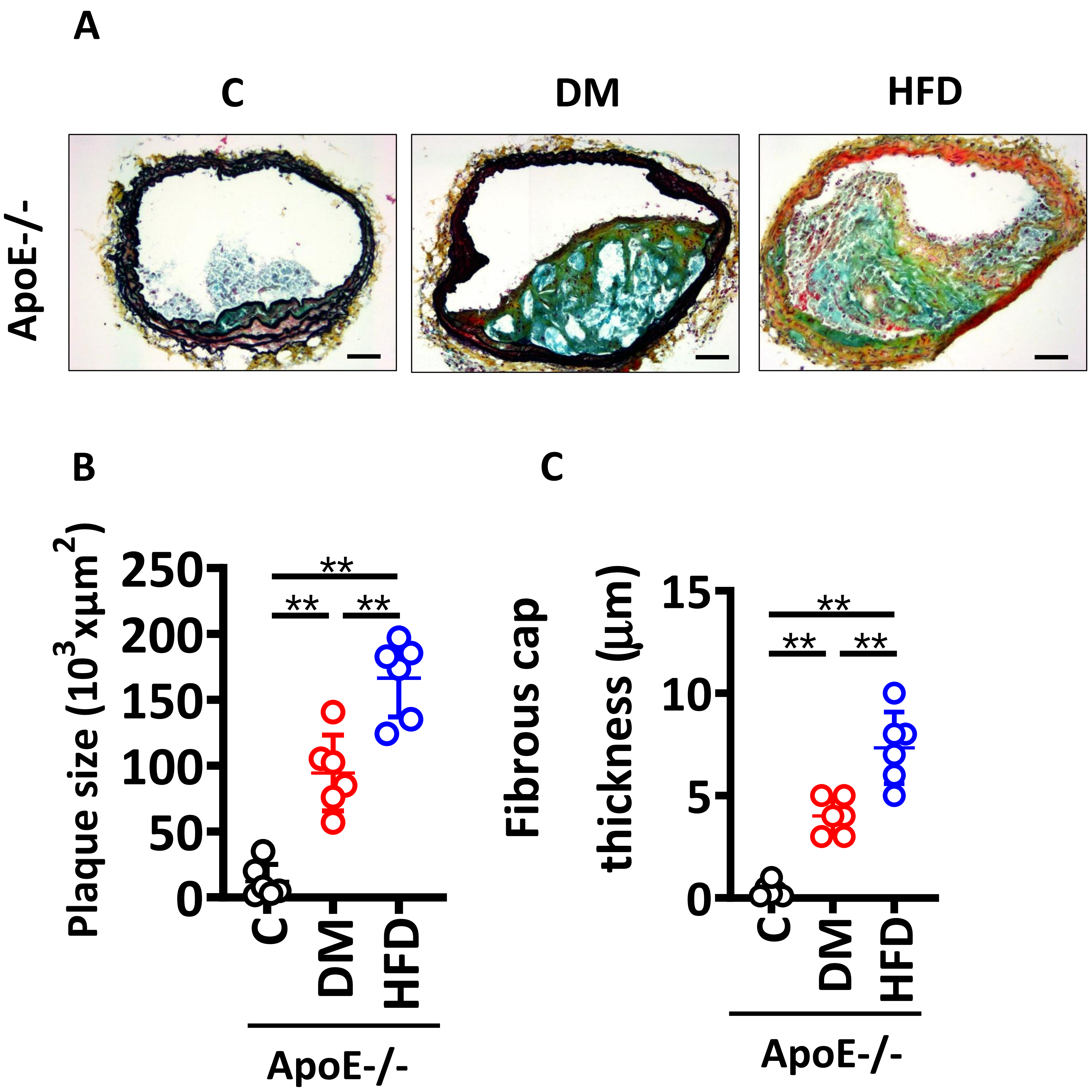

Supplementary Figure: Smaller, but less stable plaques in hyperglycaemic versus hyperlipidaemic ApoE-deficient mice. Hyperglycaemic mice have smaller plaque size compared to HFD but increased than control mice (A, B: MOVATs staining of the brachiocephalic arteries). Morphometric analysis shows a thinner fibrous cap in ApoE-/- DM vs ApoE-/- HFD mice (C). Each dot represents data obtained from one biological specimen; **P<0.01; ANOVA.
